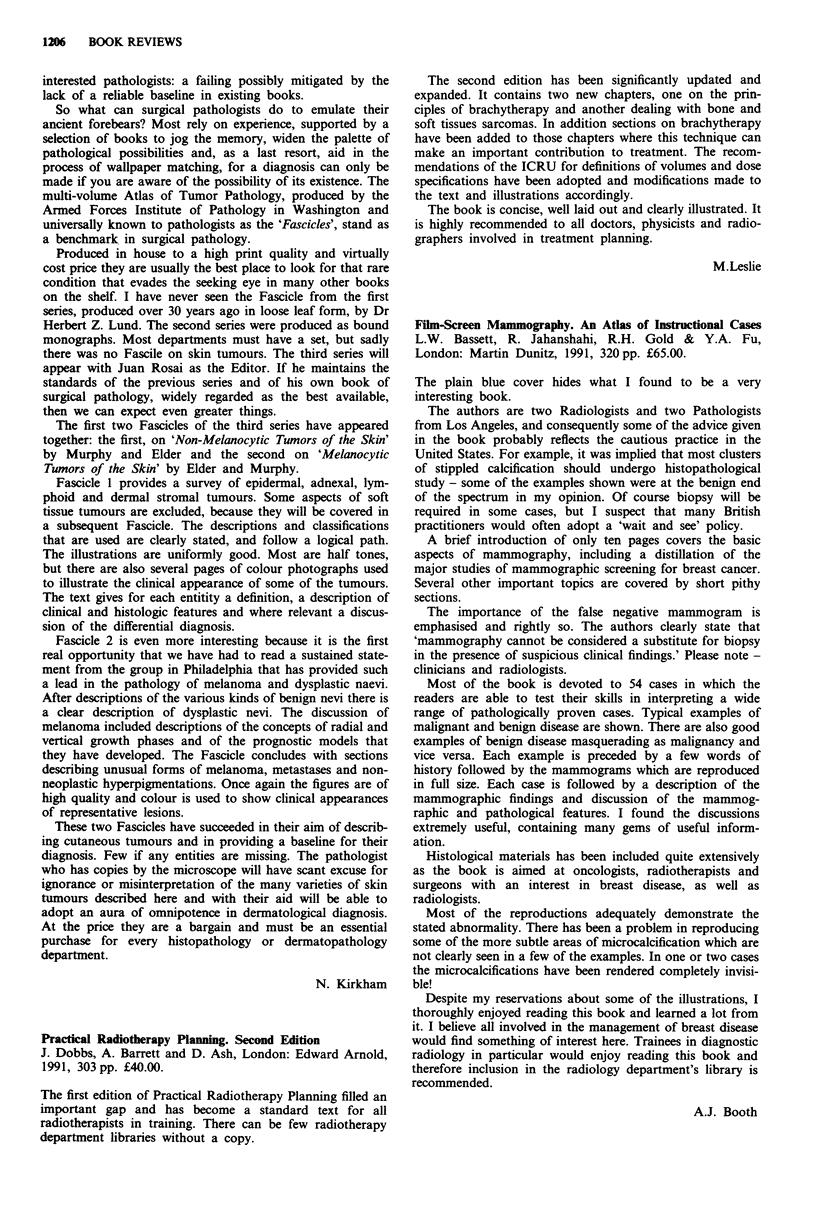# Film-Screen Mammography. An Atlas of Instructional Cases

**Published:** 1992-12

**Authors:** A.J. Booth


**Film-Screen Mammography. An Atlas of Instructional Cases**
L.W. Bassett, R. Jahanshahi, R.H. Gold & Y.A. Fu, London: Martin Dunitz, 1991, 320 pp. £65.00.

The plain blue cover hides what I found to be a very interesting book.

The authors are two Radiologists and two Pathologists from Los Angeles, and consequently some of the advice given in the book probably reflects the cautious practice in the United States. For example, it was implied that most clusters of stippled calcification should undergo histopathological study – some of the examples shown were at the benign end of the spectrum in my opinion. Of course biopsy will be required in some cases, but I suspect that many British practitioners would often adopt a 'wait and see' policy.

A brief introduction of only ten pages covers the basic aspects of mammography, including a distillation of the major studies of mammographic screening for breast cancer. Several other important topics are covered by short pithy sections.

The importance of the false negative mammogram is emphasised and rightly so. The authors clearly state that 'mammography cannot be considered a substitute for biopsy in the presence of suspicious clinical findings.' Please note – clinicians and radiologists.

Most of the book is devoted to 54 cases in which the readers are able to test their skills in interpreting a wide range of pathologically proven cases. Typical examples of malignant and benign disease are shown. There are also good examples of benign disease masquerading as malignancy and vice versa. Each example is preceded by a few words of history followed by the mammograms which are reproduced in full size. Each case is followed by a description of the mammographic findings and discussion of the mammographic and pathological features. I found the discussions extremely useful, containing many gems of useful information.

Histological materials has been included quite extensively as the book is aimed at oncologists, radiotherapists and surgeons with an interest in breast disease, as well as radiologists.

Most of the reproductions adequately demonstrate the stated abnormality. There has been a problem in reproducing some of the more subtle areas of microcalcification which are not clearly seen in a few of the examples. In one or two cases the microcalcifications have been rendered completely invisible!

Despite my reservations about some of the illustrations, I thoroughly enjoyed reading this book and learned a lot from it. I believe all involved in the management of breast disease would find something of interest here. Trainees in diagnostic radiology in particular would enjoy reading this book and therefore inclusion in the radiology department's library is recommended.

A.J. Booth